# Manila Paper Splint

**Published:** 1871-04

**Authors:** 


### MANILA PAPER-SPLINT.

We desire to call special attention to an article in this number of the Journal on manila paper, as a material for splints, by Dr. R. O. Cowling, Demonstrator of Anatomy in the University of Louisville. We have had an opportunity, within the past two months, of testing this dressing in four cases of fracture, in the lower extremity, and in one of the upper extremity.

The first case in which we applied the paper-dressing, was a fracture of the fibula, with rupture of the deltoid ligament, and were so much pleased that we determined to test it in other cases. A few days later, we saw two cases of fracture of the tibia and fibula, in both, the fracture occurring in the lower portion of the middle third. The manila paper-splint was applied in both cases, and the results were all that could be desired.

The fourth case we regard as a test of its superiority over the immovable apparatus, made with other material. This was a compound fracture of the thigh, middle third, with three points of suppuration, but one, however, communicating with the fractured bone. The fracture had been treated for six weeks, first with Smith's auterior splint, and later, with straight splints and extention by means of weights and pulley.

The patient was greatly exhausted from profuse suppuration, kept up by the constant irritation of the movable fragments. The condition of the patient was regarded as extremely critical.

The manila paper apparatus was applied in accordance with the suggestion of Dr. Cowling, and, to our great gratification, we found that it filled the indications more perfectly than was thought possible—immovably fixing the fragments, and thus, at once, greatly reducing the extensive and exhausting suppuration.

The openings in the splint, for the exit of pus, were readily made as the strips of paper were being applied, and may be made in any number, shape, size, or location, without the least difficulty. The condition of the patient rapidly improved. Five weeks after the application the splint was opened, and the position of the bone found unchanged, with a sufficient deposit of ossific matter to hold the fragments in position. The condition of the patient at present, six weeks after the dressing, is entirely satisfactoy.

The subject of the fifth case, in which we have applied the manila paper-splint, was a prominent physician of this city, Dr. J. M. Johnson who was, at one time, connected with the editorial department of this Journal. Fifteen days ago the Doctor's horse took fright, became unmanagable and threw him from his buggy, inflicting a serious injury in the vicinity of the right shoulder joint. As usual, when a physician is the patient, an examination sufficiently rigid to determine the precise character of the injury, was not permitted.

In a few hours the shoulder was considerably swollen, the least movement of the arm producing the most acute pain. Various plans to support and fix the arm were suggested and applied, but proved ineffectual in securing the desired comfort.

For twelve days and nights this state of things continued, the Doctor, during this time, sitting or reclining in an invalid-chair, fixing the injured arm with the opposite hand, or by pillows placed upon a stand, erected by his chair. We, with others, had several times suggested the immovable apparatus as likely to give him rest from his watching, but not until he was greatly exhausted from pain, position, etc., would he consent to its application. As the swelling subsided, which it did to a considerable extent in eight or ten days, the rational signs of fracture of the surgical neck of the humorous were much more manifest. He still refused, however, to submit to any further tests, cutting short any argument in that direction with the emphatic no !

On the twelfth day after the injury, the manila paper-dressing was applied, as illustrated in electrotype, Fig. 4, Dr. Cowling's article. In a few hours the Doctor was more comfortable. The day after, upon calling, we found that he had left his invalid-chair; and to our surprise and gatification, we met him this morning, two days after the dressing, in his office prescribing for a patient, and certainly, greatly pleased with the manila paper-dressing.

